# Supplementary material for: Concomitant phytonutrient and transcriptome analysis of mature fruit and leaf tissues of tomato (Solanum lycopersicum L. cv. Oregon Spring) grown using organic and conventional fertilizer
Source: PLoS One. 2020 Jan 13;15(1):e0227429. doi: 10.1371/journal.pone.0227429 (PMC6957345; doi:10.1371/journal.pone.0227429)
Supplement: S1 Table — (DOCX) [file pone.0227429.s001.docx]

Supplementary Table 1: Above (vine) and below ground (root) vegetative biomass on fresh weight (FW) and dry weight (DW) bases, and percent root biomass fraction for conventional (CONV) and organic (ORG) fertilizer treatments. Data were analyzed using ANOVA General Linear Model. (See Supplementary file for measured observations, LS means, standard deviations, and standard errors.)

| Biomass | Units | Main Effect Means | |
| --- | --- | --- | --- |
|  |  | CONV | ORG |
| Above ground (vine) FW | kg | 1.32 | 1.18^NS^ |
| Below ground (root) FW | g | 36.4 | 35.1^NS^ |
| Above ground (vine) DW | g | 115 | 110^NS^ |
| Below ground (root) DW | g | 4.90 | 5.20^NS^ |
| Root fraction DW | % | 4.19 | 4.71^NS^ |

^NS^Non-significant
