# Supplementary material for: Concomitant phytonutrient and transcriptome analysis of mature fruit and leaf tissues of tomato (Solanum lycopersicum L. cv. Oregon Spring) grown using organic and conventional fertilizer
Source: PLoS One. 2020 Jan 13;15(1):e0227429. doi: 10.1371/journal.pone.0227429 (PMC6957345; doi:10.1371/journal.pone.0227429)
Supplement: S2 Table — (DOCX) [file pone.0227429.s002.docx]

Supplementary Table 2: Leaf dry matter and mineral concentrations on a dry weight (DW) basis for conventional (CONV) and organic (ORG) fertilizer treatments. Data were analyzed using ANOVA General Linear Model. (See Supplementary file for measured observations, LS means, standard deviations, and standard errors.)

|  | Units | Main Effect Means |  |
| --- | --- | --- | --- |
|  |  | CONV | ORG |
| Dry Matter | % | 8.78 | 9.41* |
| Carbon | % DW | 38.3 | 34.8*** |
| Nitrogen | % DW | 5.2 | 4.2*** |
| Carbon:Nitrogen | μg/g DW | 7.44 | 8.41** |
| Phosphorus | μg/g DW | 14,167 | 11,833* |
| Potassium | μg/g DW | 58,500 | 64,500^NS^ |
| Calcium | μg/g DW | 39,500 | 47,000** |
| Magnesium | μg/g DW | 8,467 | 9,750* |
| Sulfur | μg/g DW | 16,167 | 36,000*** |
| Sodium | μg/g DW | 975 | 930^NS^ |

*p≤0.05, **p≤0.01, p≤0.001, ^NS^non-significant
