# Supplementary figures and images for: Concomitant phytonutrient and transcriptome analysis of mature fruit and leaf tissues of tomato (Solanum lycopersicum L. cv. Oregon Spring) grown using organic and conventional fertilizer
Source: PLoS One. 2020 Jan 13;15(1):e0227429. doi: 10.1371/journal.pone.0227429 (PMC6957345; doi:10.1371/journal.pone.0227429)

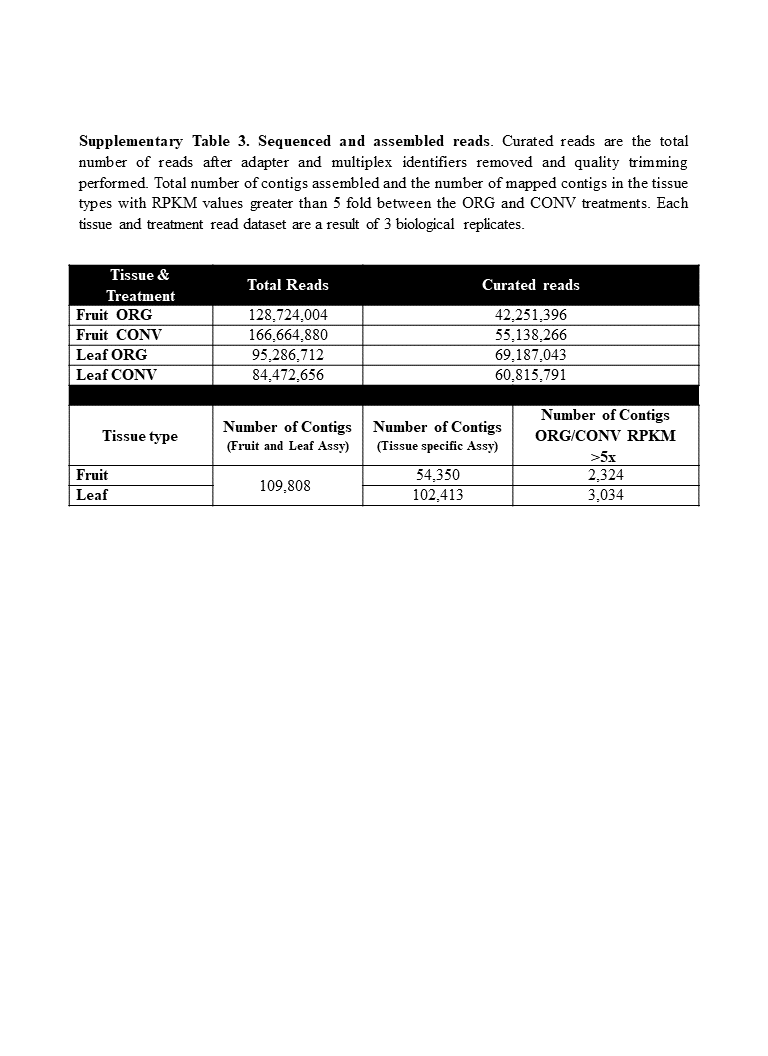

Supplement: S3 Table — (GIF) [file pone.0227429.s003.GIF]

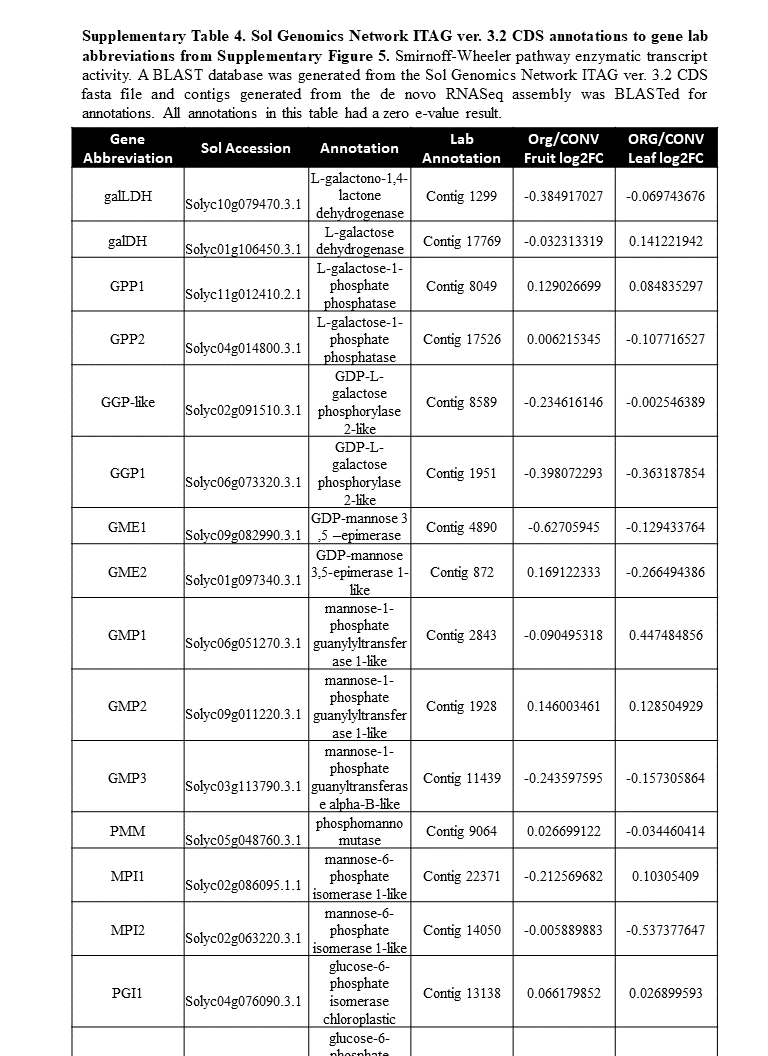

Supplement: S4 Table — (GIF) [file pone.0227429.s004.GIF]

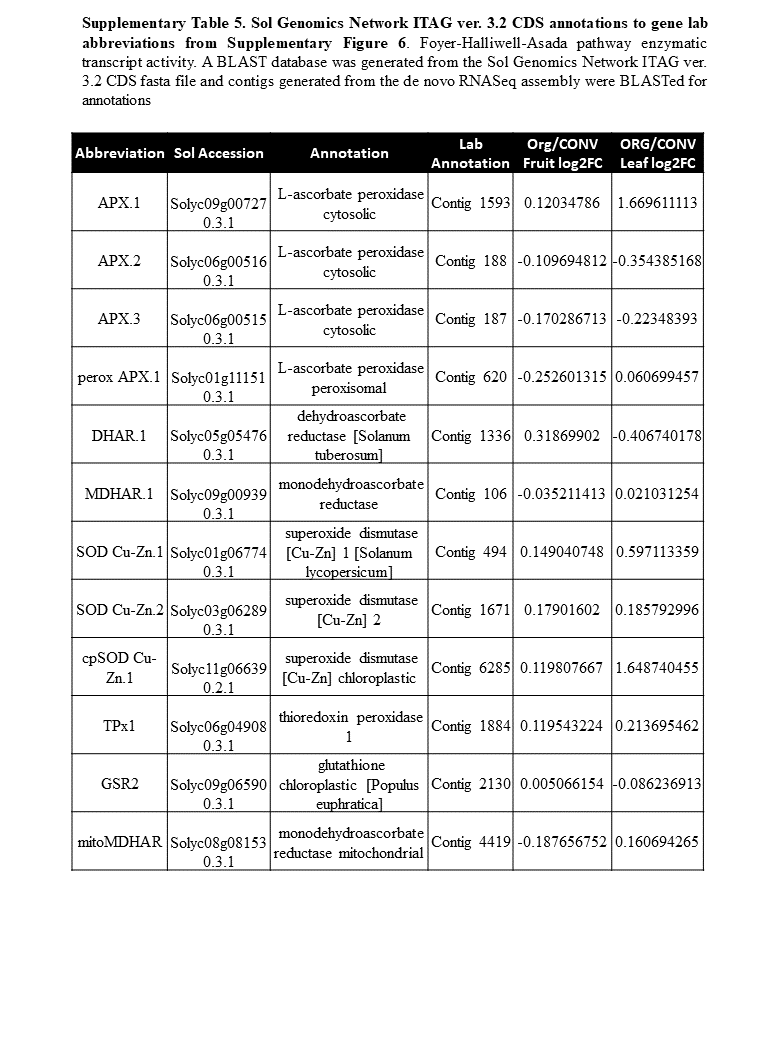

Supplement: S5 Table — (GIF) [file pone.0227429.s005.GIF]

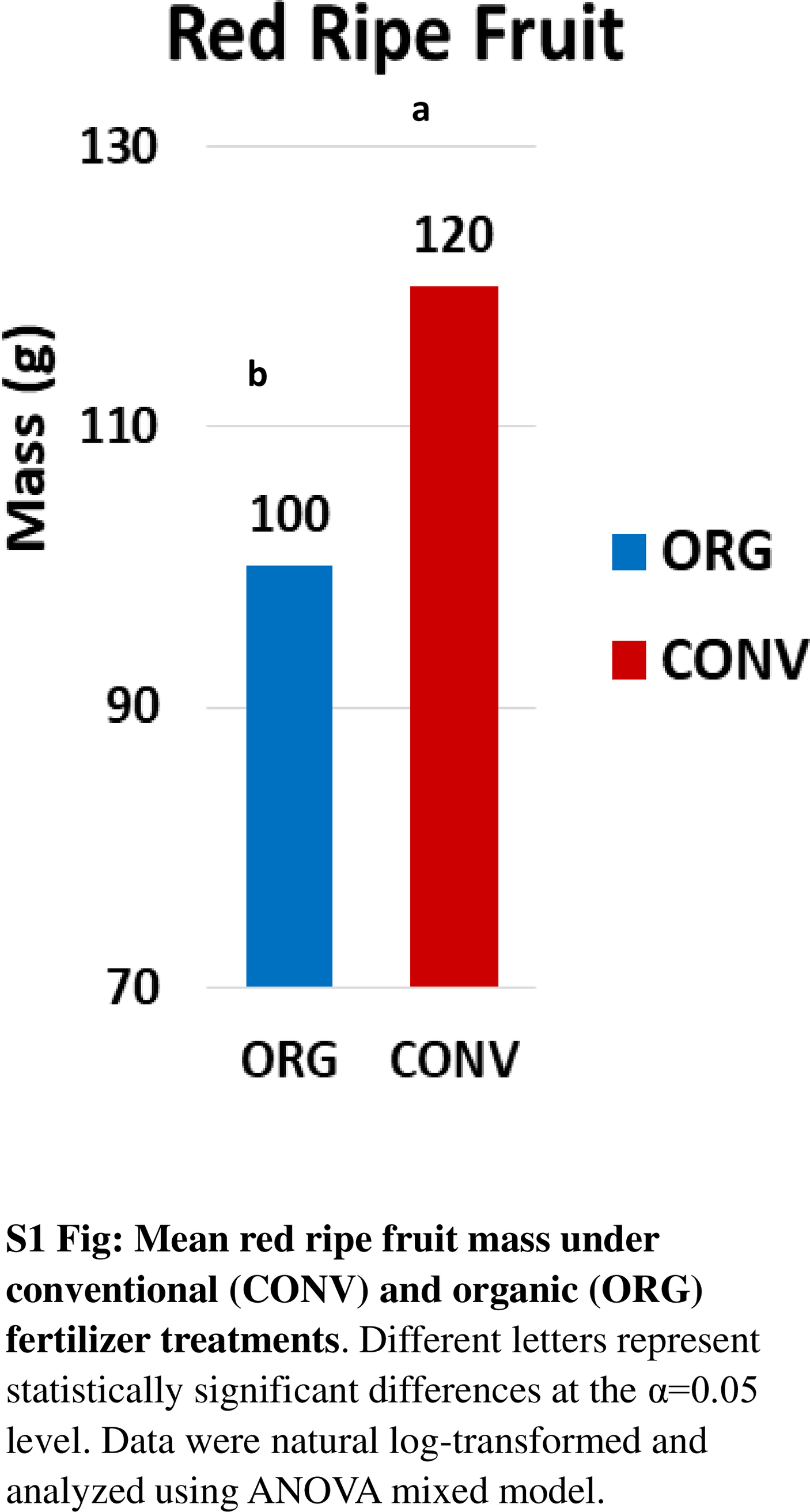

Supplement: S1 Fig — (TIF) [file pone.0227429.s006.tif]

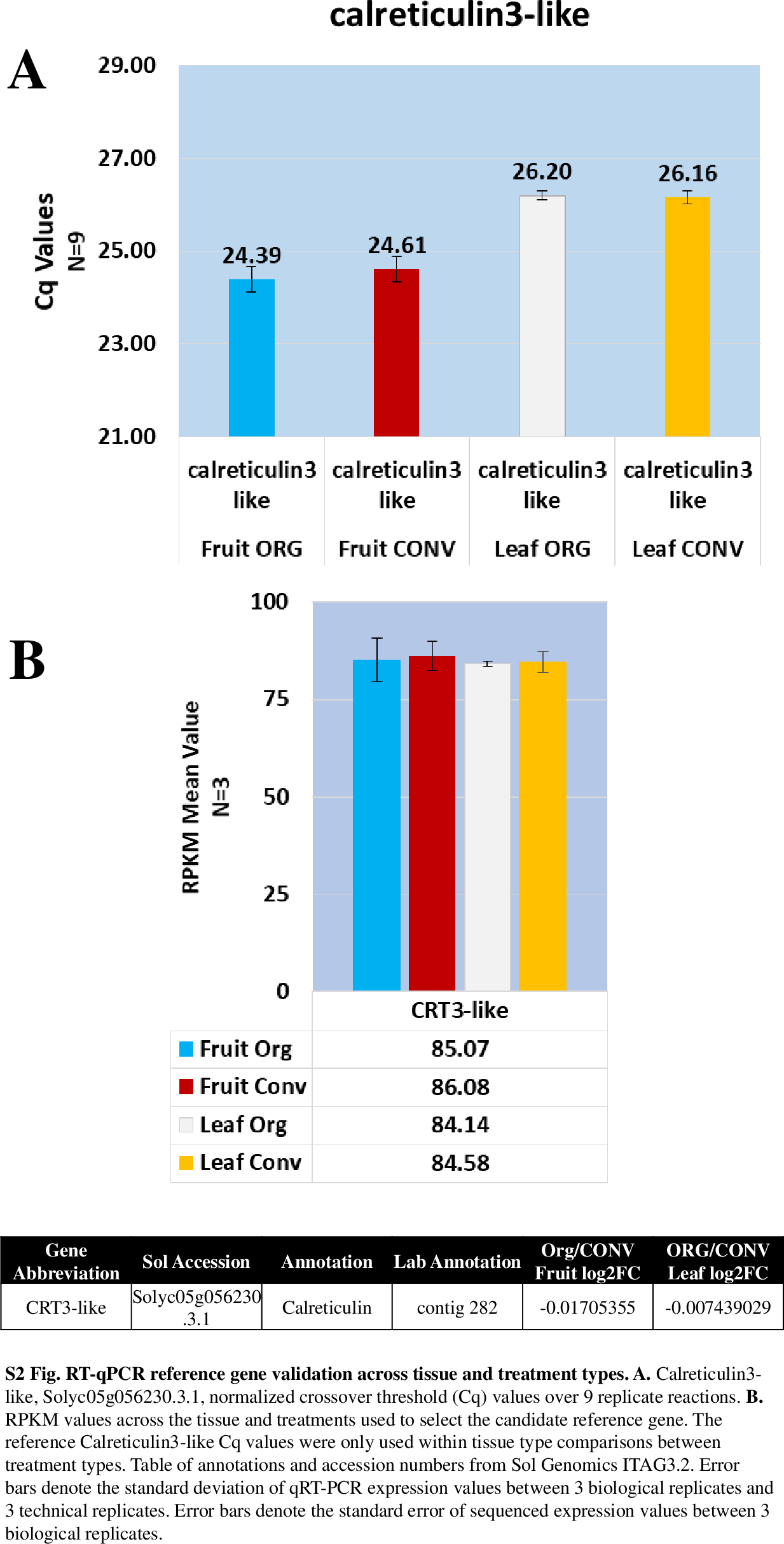

Supplement: S2 Fig — (TIF) [file pone.0227429.s007.tif]

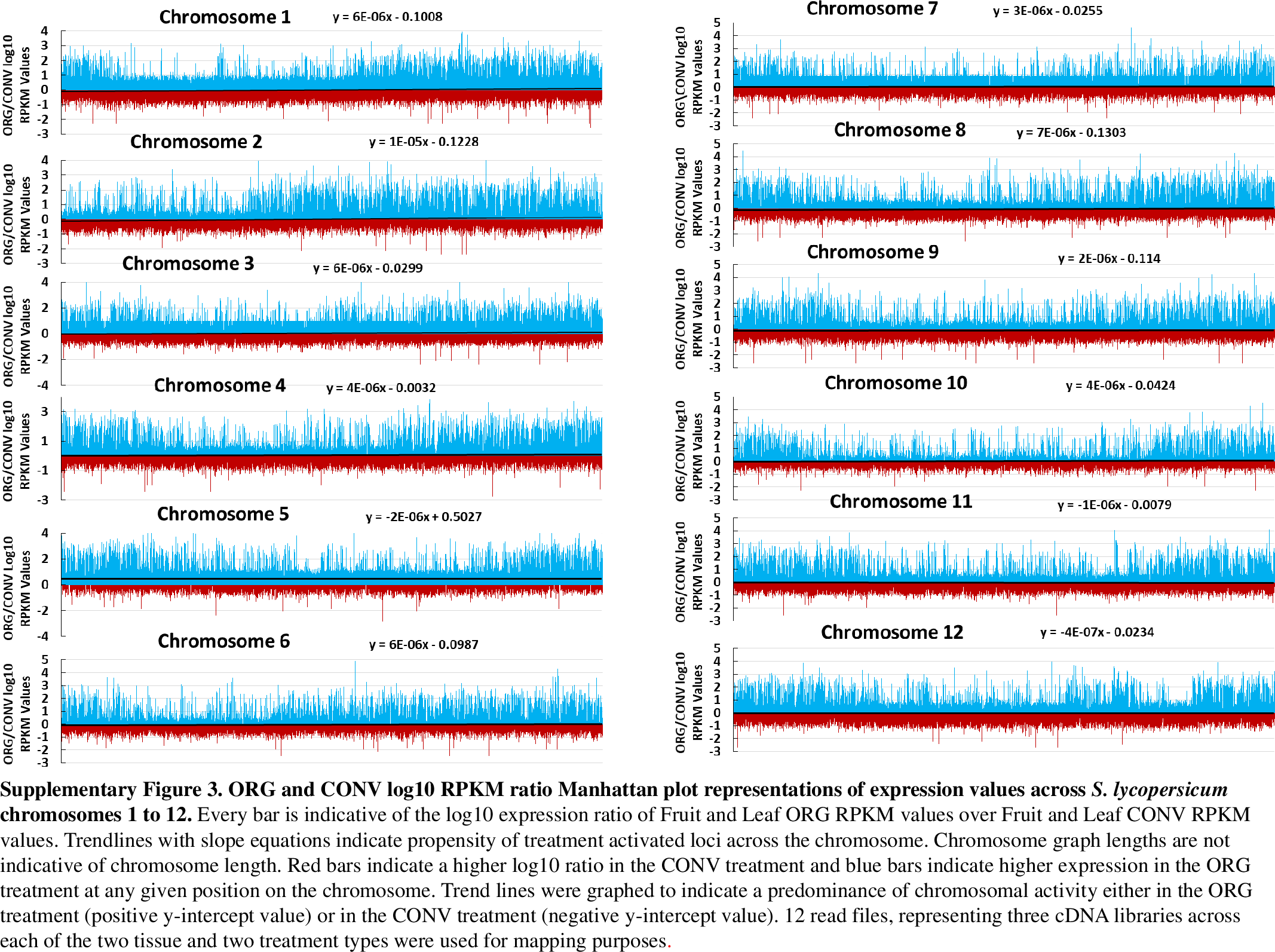

Supplement: S3 Fig — (TIF) [file pone.0227429.s008.tif]

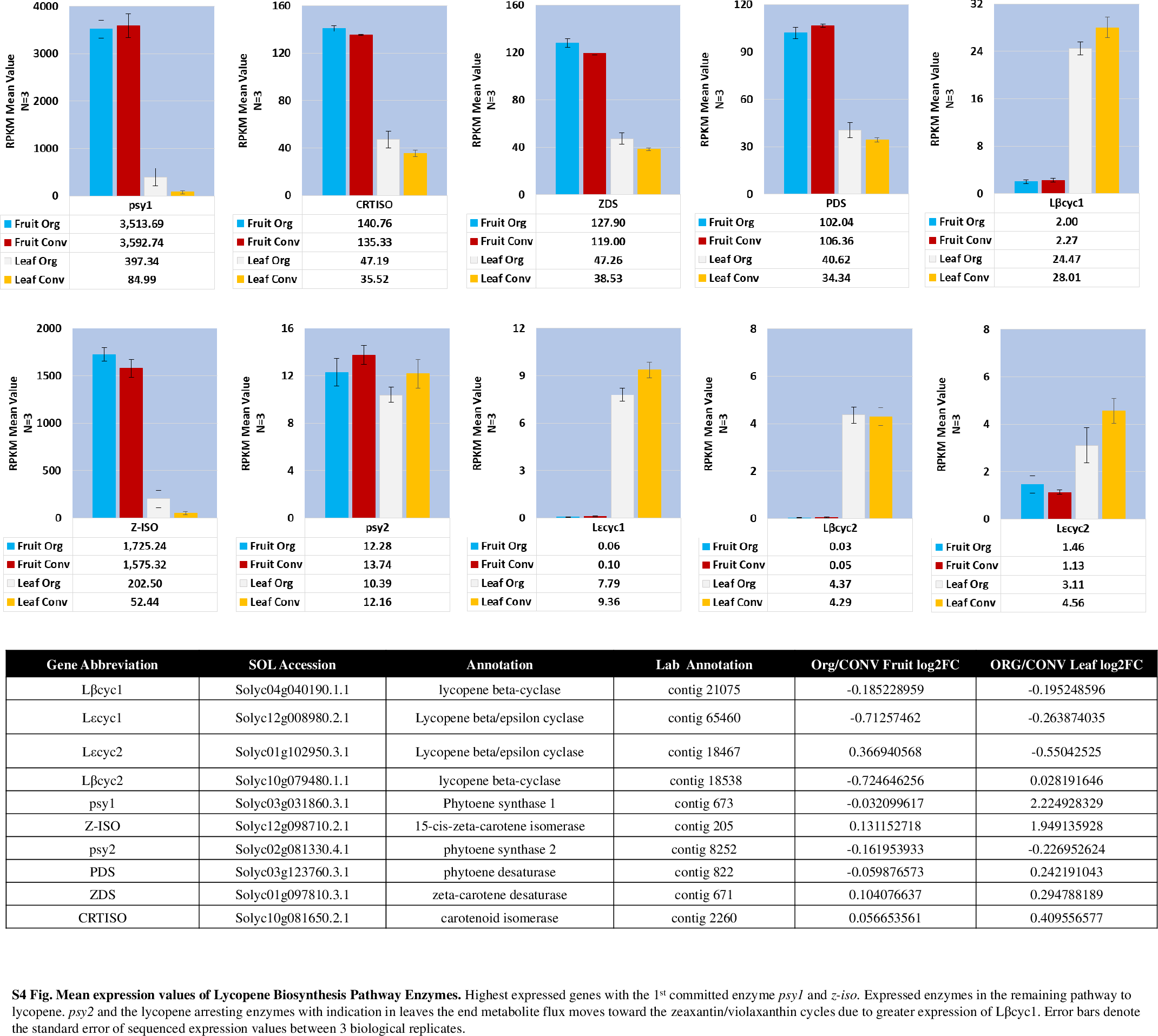

Supplement: S4 Fig — (TIF) [file pone.0227429.s009.tif]

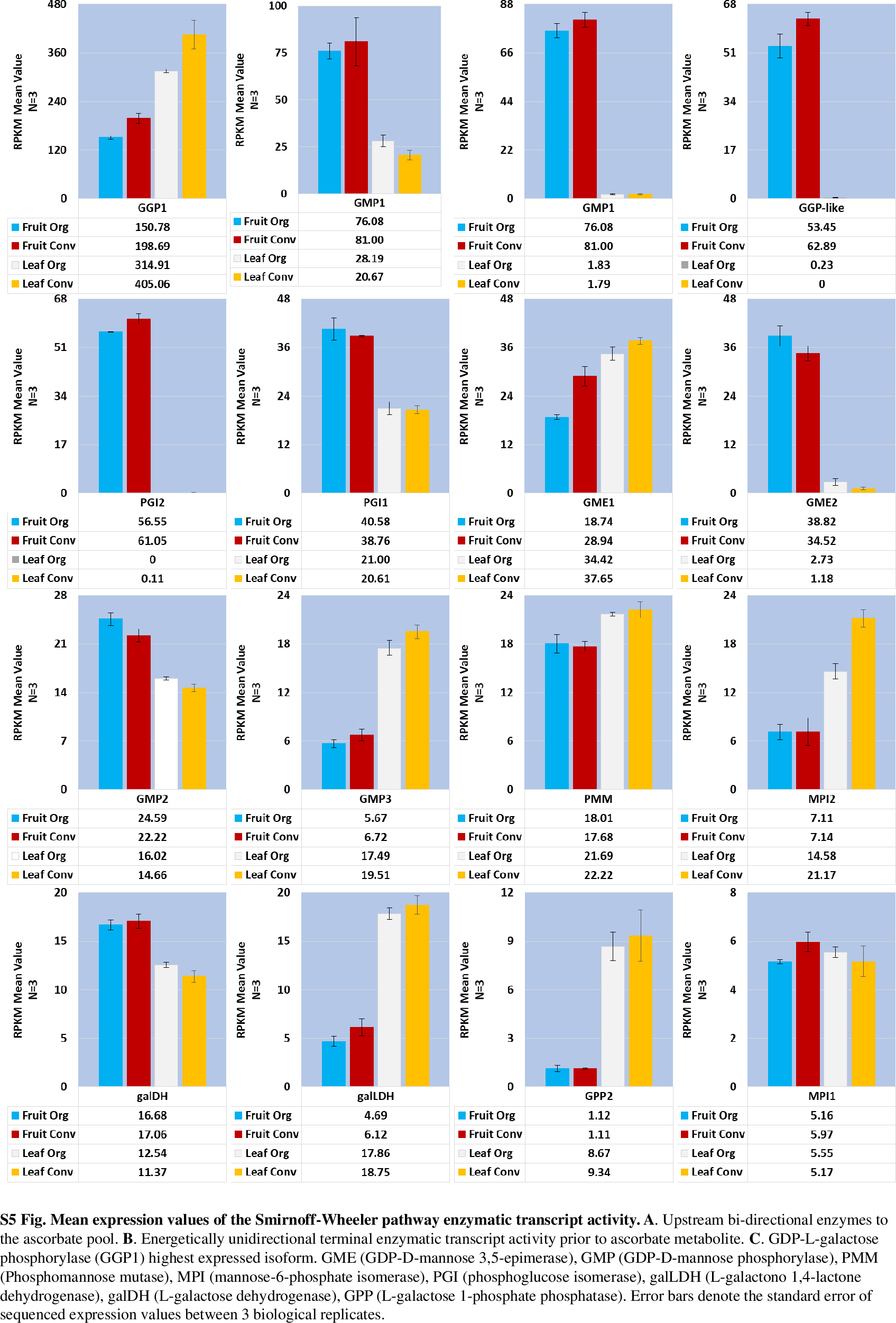

Supplement: S5 Fig — (TIF) [file pone.0227429.s010.tif]

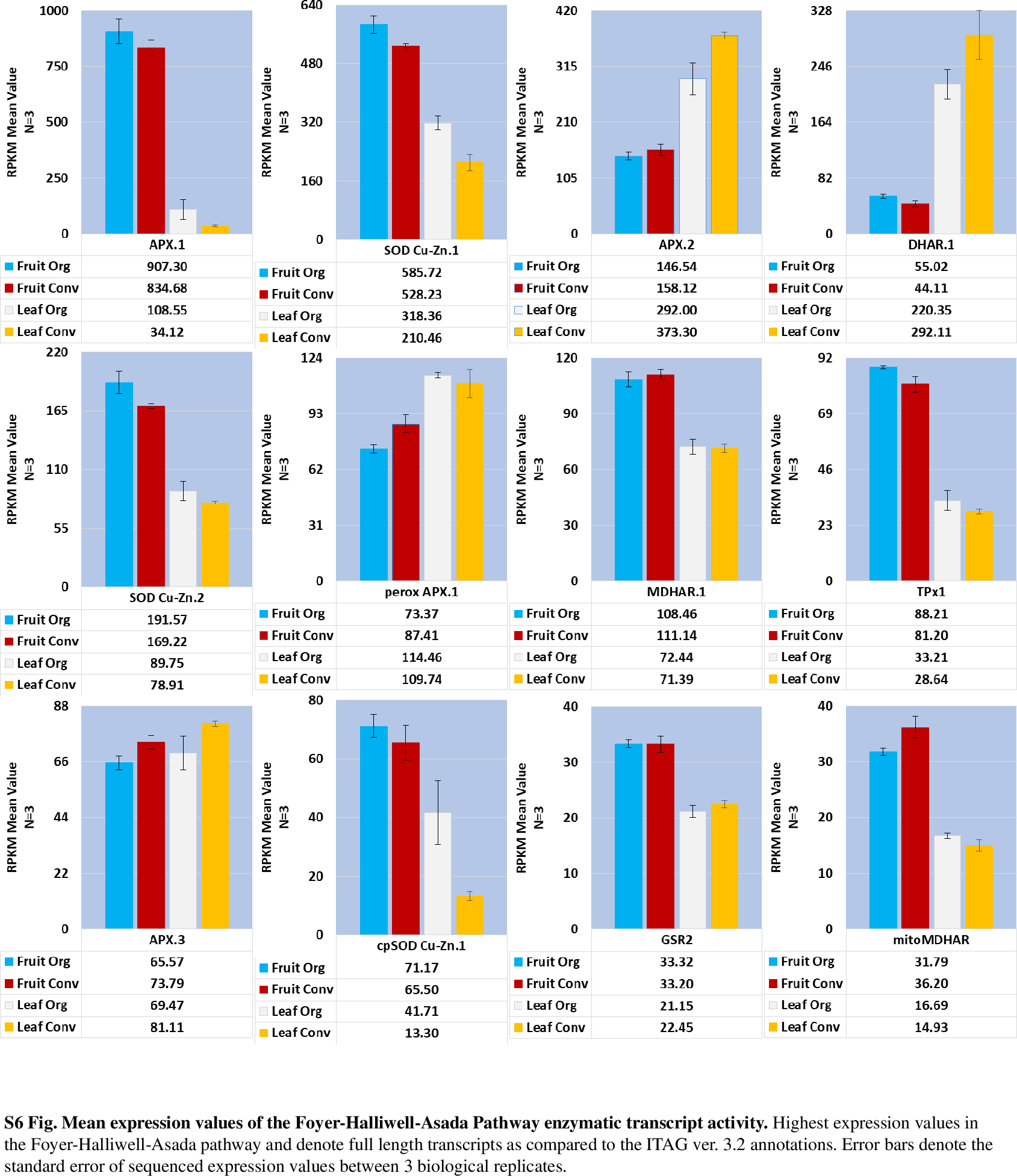

Supplement: S6 Fig — (TIF) [file pone.0227429.s011.tif]

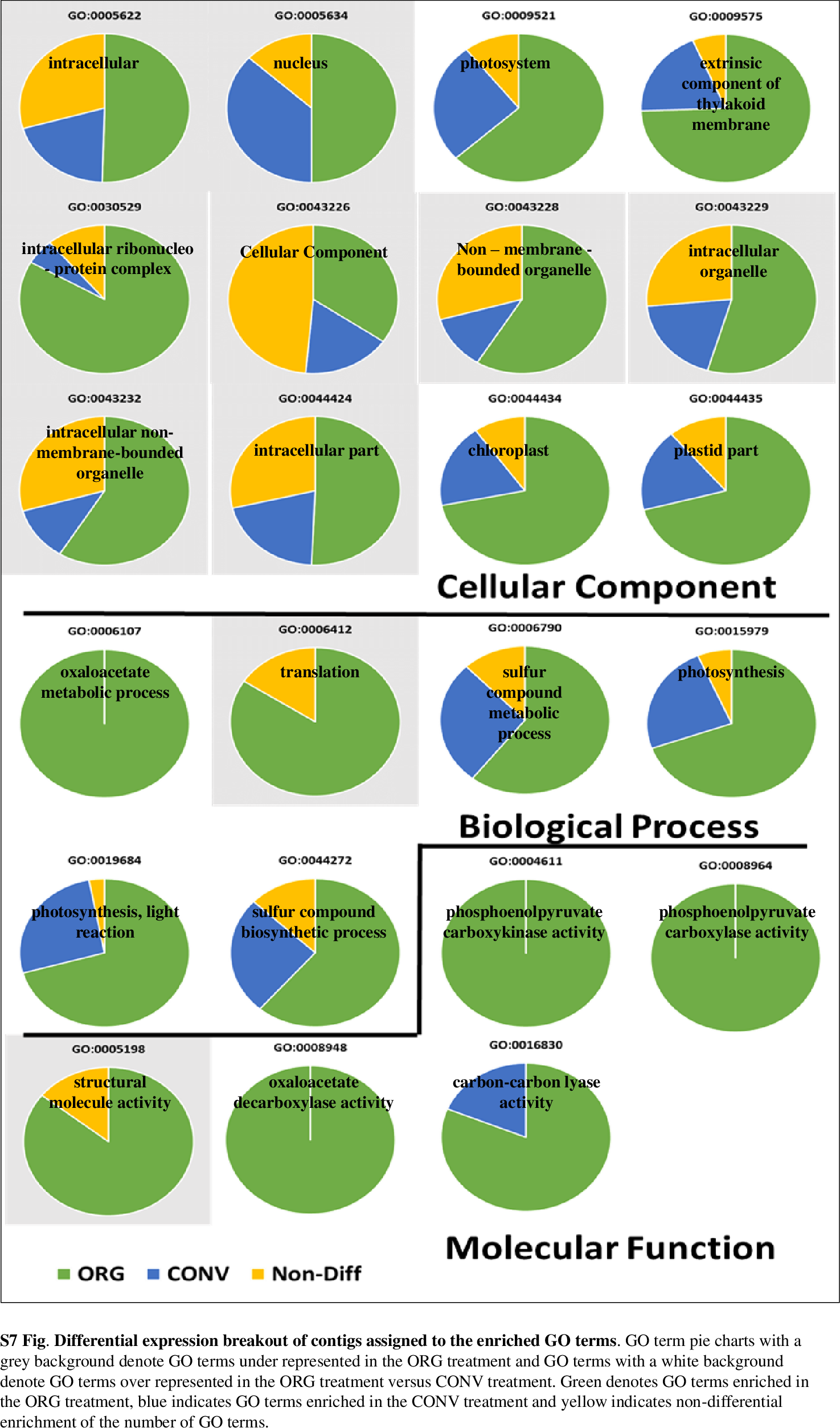

Supplement: S7 Fig — (TIF) [file pone.0227429.s012.tif]

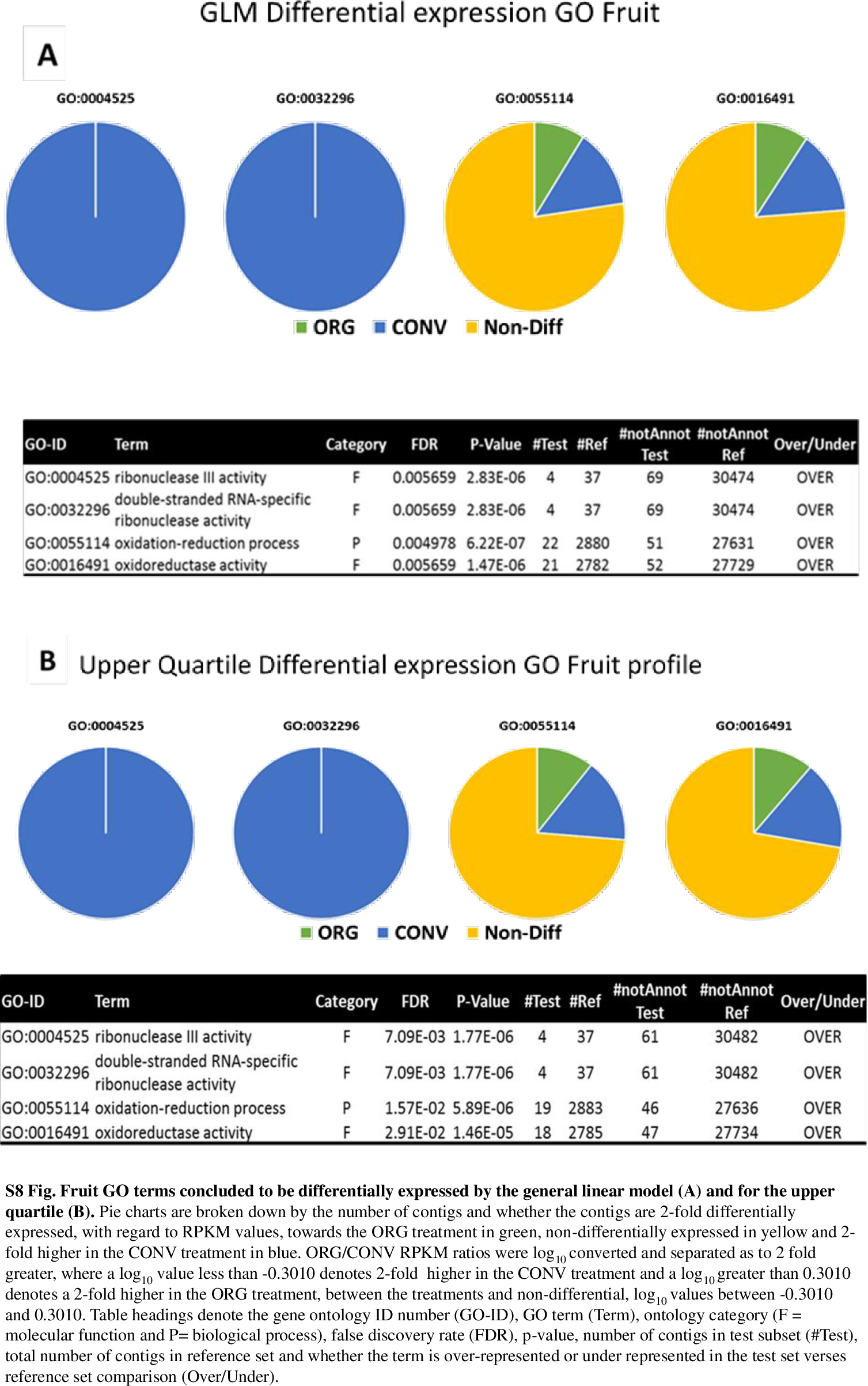

Supplement: S8 Fig — Fruit GO terms concluded to be differentially expressed by the general linear model (A) and for the upper quartile (B). (TIF) [file pone.0227429.s013.tif]

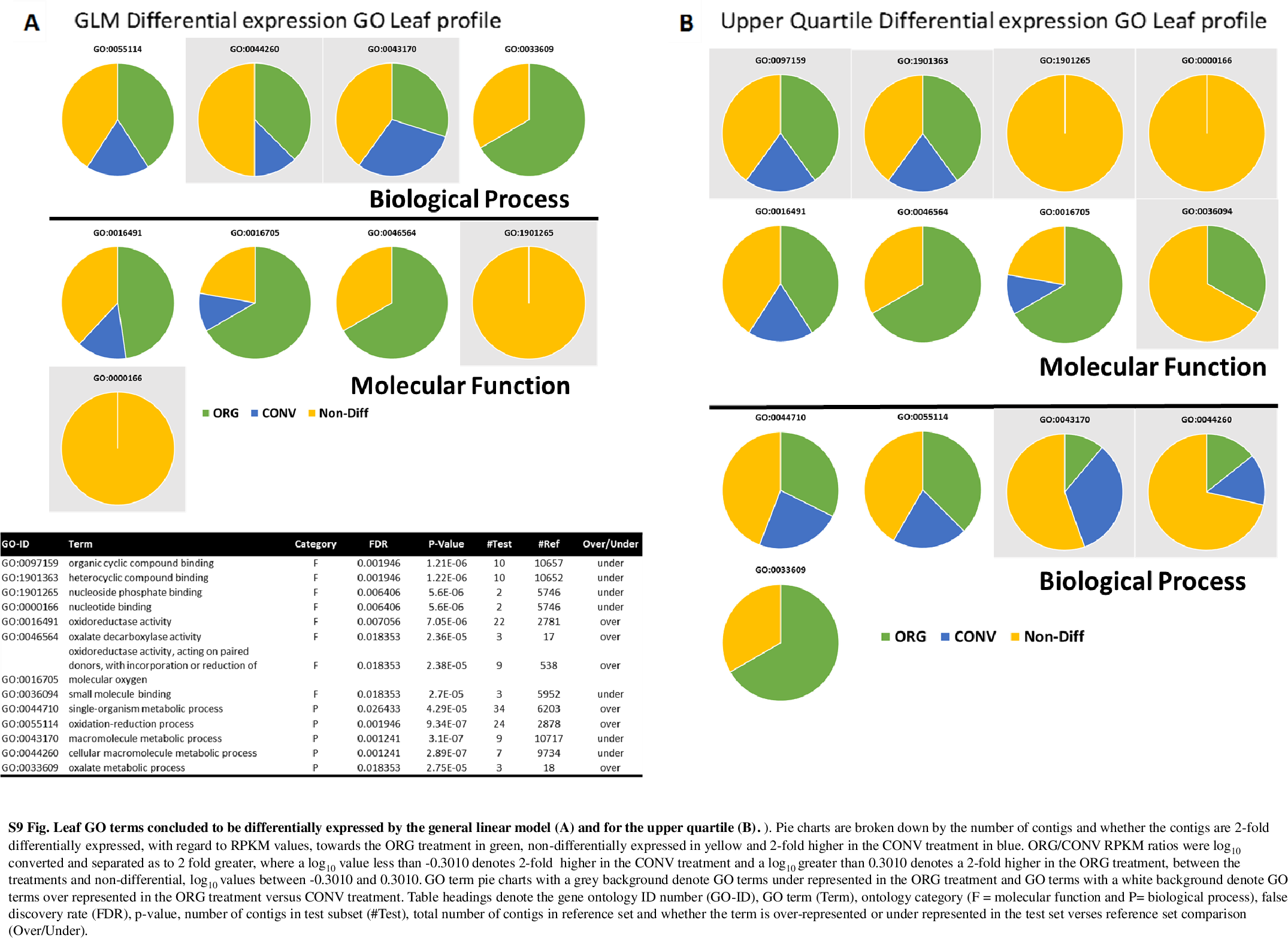

Supplement: S9 Fig — Leaf GO terms concluded to be differentially expressed by the general linear model (A) and for the upper quartile (B). (TIF) [file pone.0227429.s014.tif]

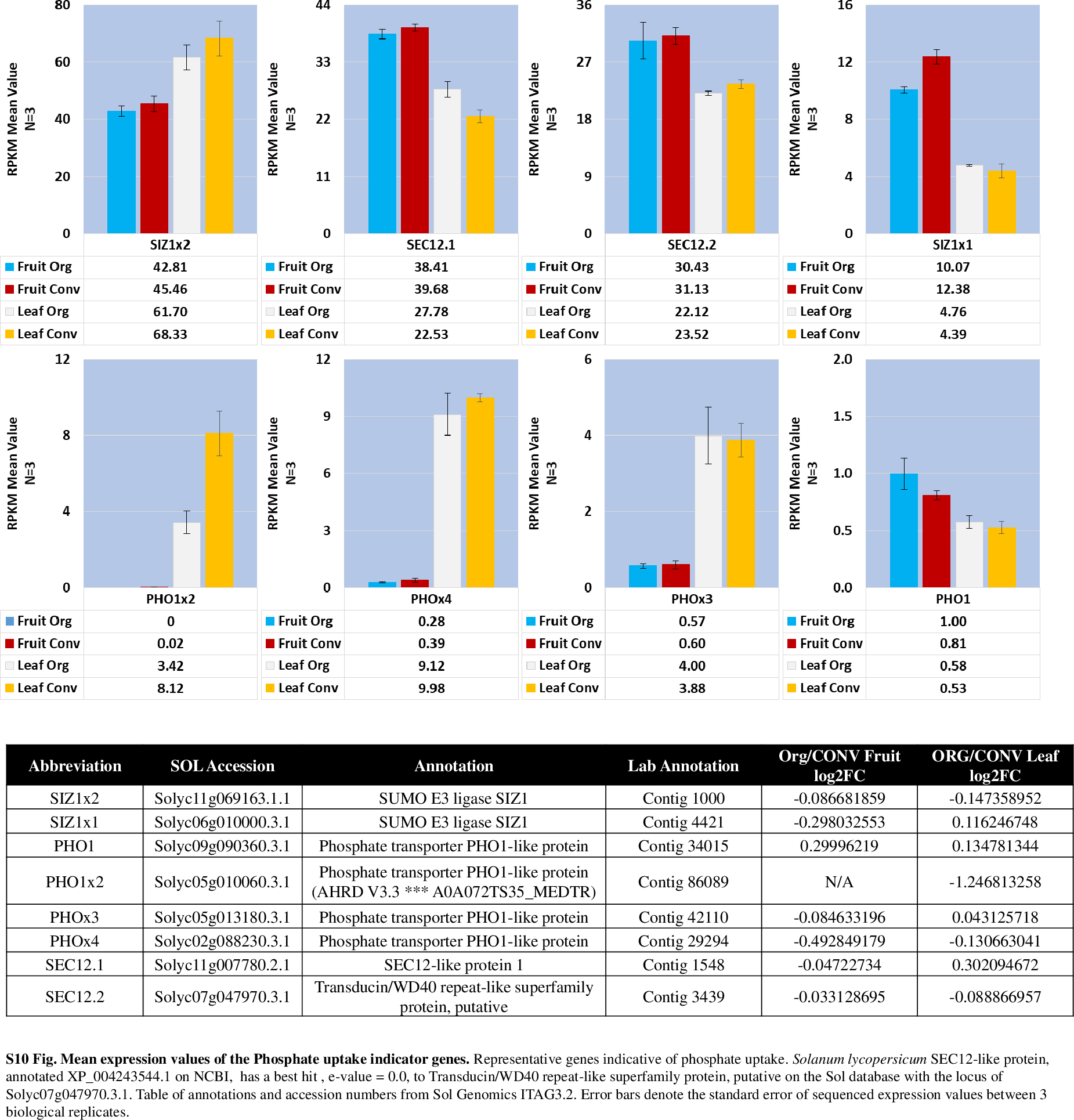

Supplement: S10 Fig — (TIF) [file pone.0227429.s015.tif]

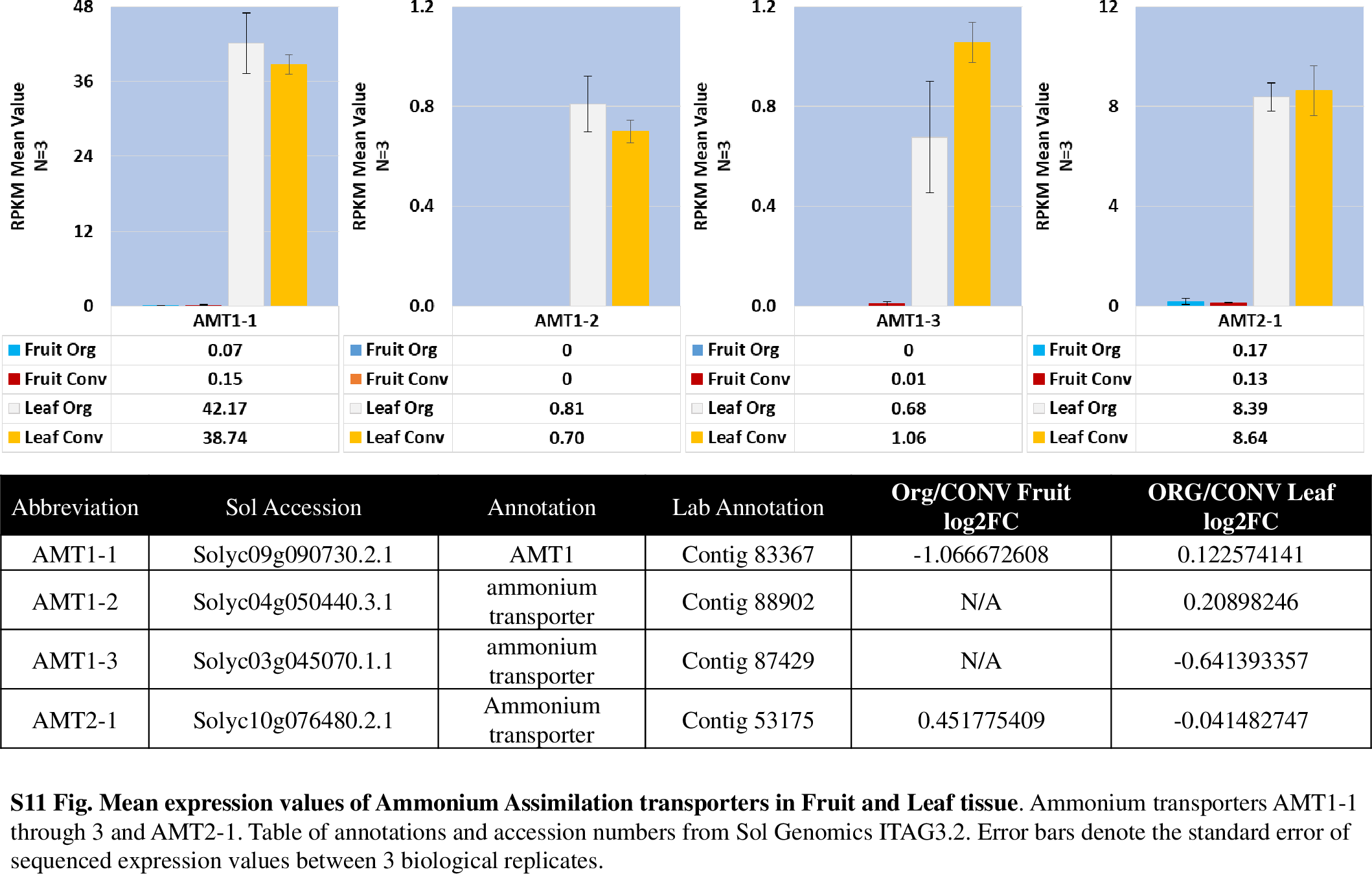

Supplement: S11 Fig — (TIF) [file pone.0227429.s016.tif]

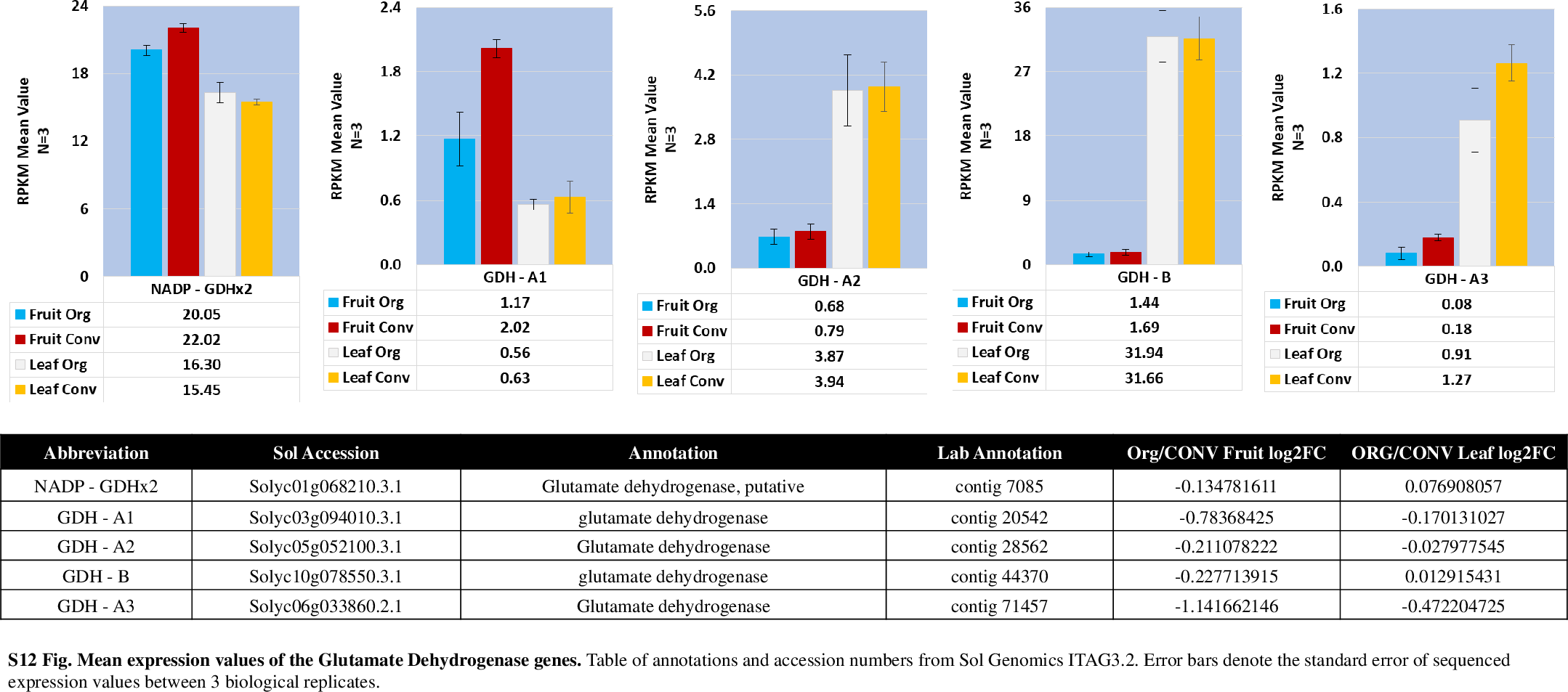

Supplement: S12 Fig — (TIF) [file pone.0227429.s017.tif]

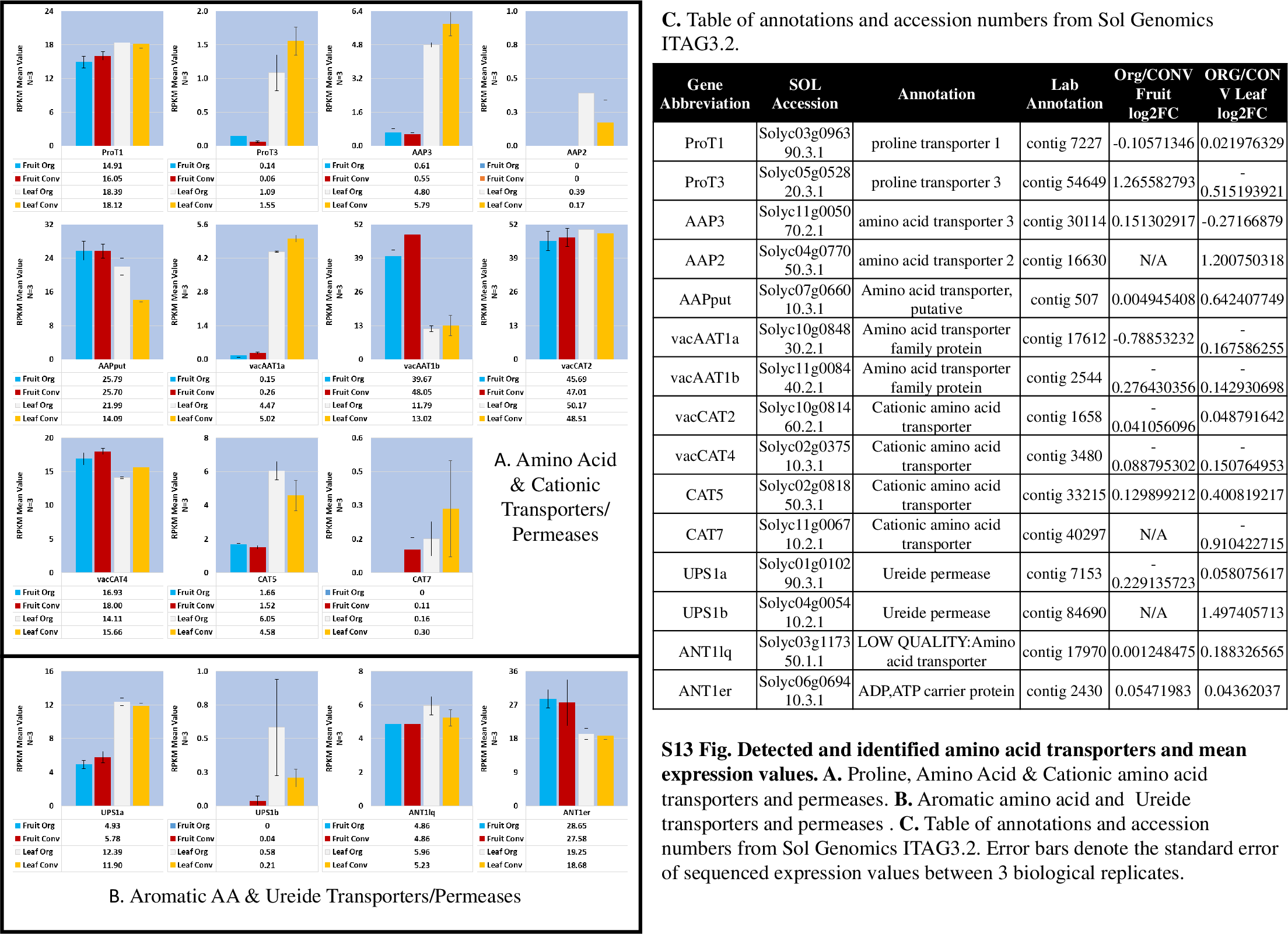

Supplement: S13 Fig — (TIF) [file pone.0227429.s018.tif]

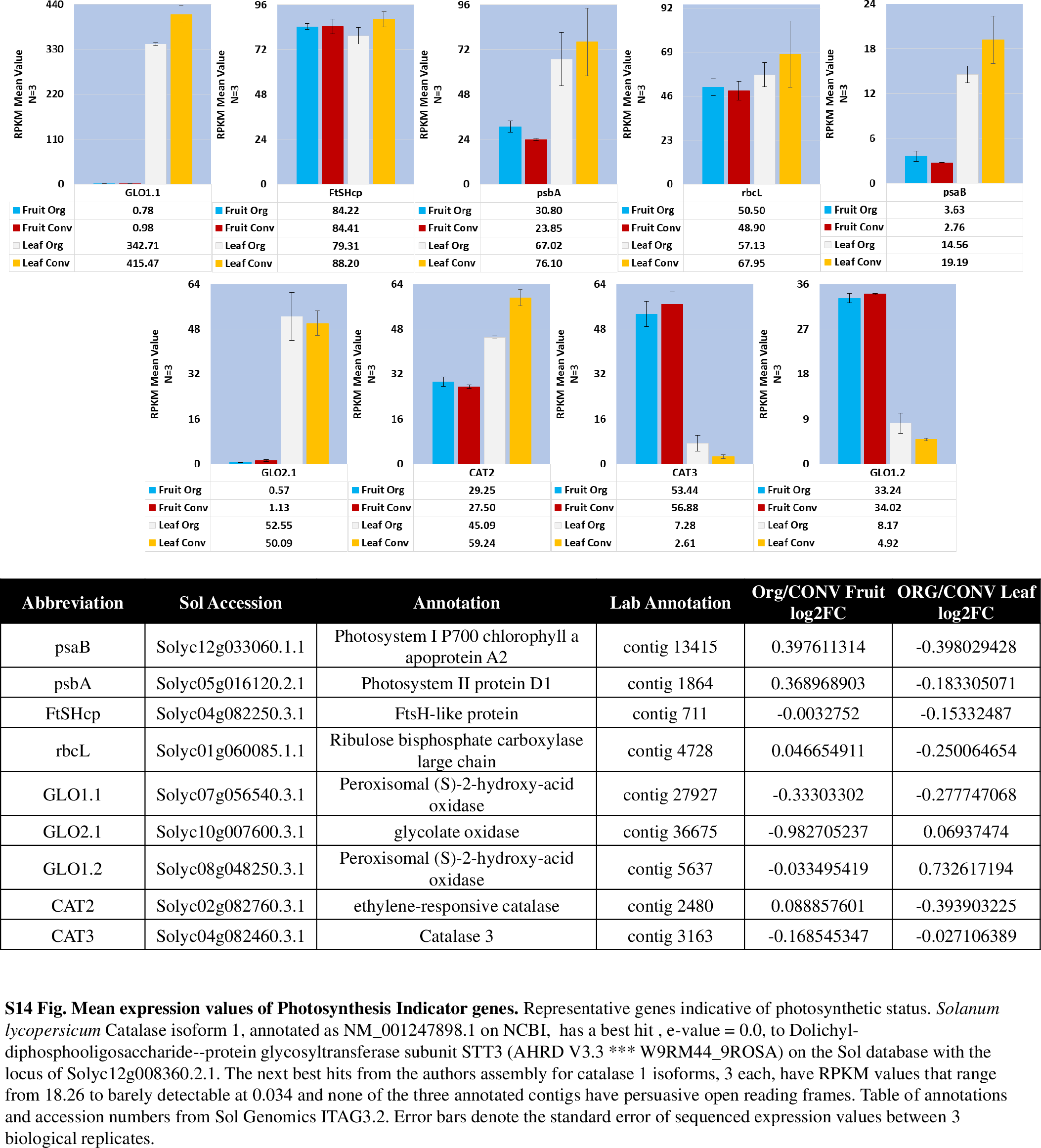

Supplement: S14 Fig — (TIF) [file pone.0227429.s019.tif]

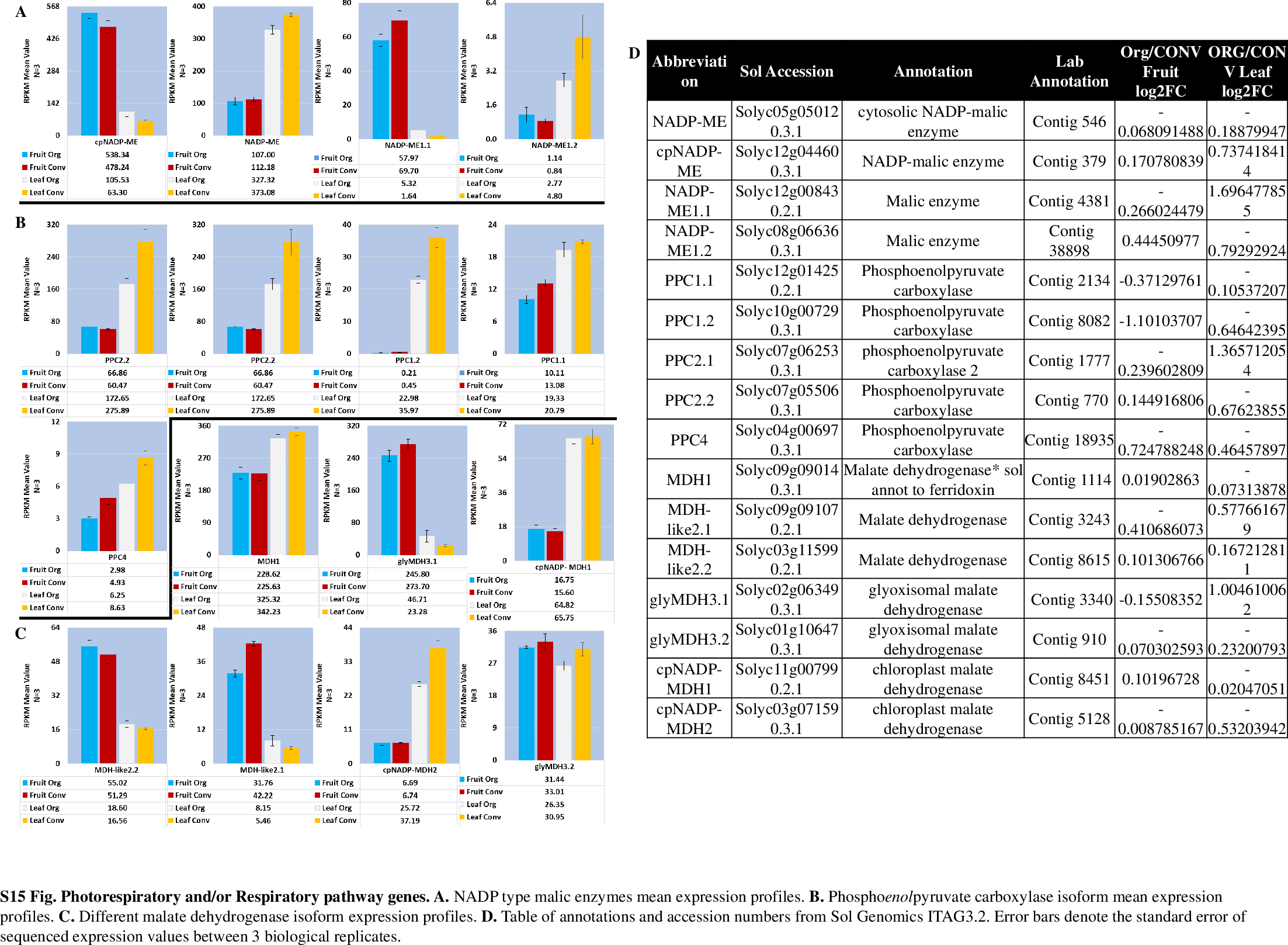

Supplement: S15 Fig — (TIF) [file pone.0227429.s020.tif]
