# Supplementary material for: Concomitant phytonutrient and transcriptome analysis of mature fruit and leaf tissues of tomato (Solanum lycopersicum L. cv. Oregon Spring) grown using organic and conventional fertilizer
Source: PLoS One. 2020 Jan 13;15(1):e0227429. doi: 10.1371/journal.pone.0227429 (PMC6957345; doi:10.1371/journal.pone.0227429)
Supplement: Attachment — Submitted filename: ReviewerCommentsAndResponses PLOS ONE_10272019.docx [file pone.0227429.s021.docx]

Dear Editor,

The authors are thankful to the reviewers for their critical and supportive comments. We have considered each comment and have made revisions to the manuscript along with responding to the comments. A point-by-point response to the reviewer’s comments is provided below. The suggested revisions have improved the manuscript and we hope that the manuscript will now be acceptable for publication in PLOS One.

**Editorial Comment:** We note that you have included the phrase “data not shown” in your manuscript. Unfortunately, this does not meet our data sharing requirements. PLOS does not permit references to inaccessible data. We require that authors provide all relevant data within the paper, Supporting Information files, or in an acceptable, public repository. Please add a citation to support this phrase or upload the data that corresponds with these findings to a stable repository (such as Figshare or Dryad) and provide and URLs, DOIs, or accession numbers that may be used to access these data. Or, if the data are not a core part of the research being presented in your study, we ask that you remove the phrase that refers to these data.
**Response**: The phrase “data not shown” was an oversight in the original submission. All data are included in the main body or in the supplementary section of the revised submission.

**Comment #1**: This manuscript presenting some valuable information and has sufficient level of novelty, this may be published after necessary revision.

**Response**: Authors appreciate the reviewer’s overall support.

**Comment #2**: Page 7: “In this study, less than a 9% difference in the transcriptional abundance of GPP1 between the fruit tissues was observed” Normally differential expression is mentioned based on Log2 fold change, you have calculated the difference of expression in percentages, apparently 9% seems an obvious change however if you calculate based on log2 fold change the difference might not be so obvious, please check correct accordingly. Please represent the changes (up-regulation and down-regulation) in gene expressions/transcripts based on log2 fold change rather fold change or percentages (throughout the document).

**Response**: The authors agree with the reviewer that normally differential expression is mentioned based on log2 fold-change. The focus of this study, as stated in the hypothesis, was that the genes involved in pathways that corresponded to the phytonutrient differences (Lycopene and Ascorbate pathways), would be differentially expressed. However, we didn’t find support for the hypothesis, perhaps due to the fact that we analyzed only the mature leaf and fruit tissues.

When we performed GO (gene ontology) enrichment, we observed the terms that were differentially enriched between ORG vs CONV. In the ontologies that showed significant enrichment according to the Fisher’s Exact Test, we identified the associated genes and their RPKM values. Thereafter, we analyzed the changes in RPKM values of the said genes in the context of the pathways they participate in. This methodology is detailed in the methods section. We refrained from stating that any of these genes were significantly differentially expressed unless the RPKM values were doubled from one treatment to the other, which then would be considered to be differentially expressed using the log2 fold-change parameter.

We used this approach to understand the relationships between the pathway enzymes. Therefore, the data are represented as percentage based on RPKM values. However, if the editor considers we should represent the data in terms of significant changes only, we will be happy to make the necessary edits.

**Comment #3**: Page 5: Please replace “fertility treatments” with “fertilizer treatment”

**Response**: The manuscript has been edited as advised.

**Comment #4**: The manuscript seems overly long, please make it comparatively shorter.
**Response**: The authors chose to submit the manuscript to PLOS One as there are no strict limits to the length of the article. The authors contend that a study and analysis as complex as the one presented in this study warrants addressing the key pathways known to be impacted by the conditions of the study. We appreciate the reviewer’s comment, however, in the absence of specific feedback, we are unsure which sections to move to supplementary section.

**Comment #5**: Another observation is that throughout the manuscript reader has focused to highlight or show the up-regulated genes however the information or description of down-regulated genes has not got due attention.
**Response**: This is indeed an astute observation, something the authors had noticed during the preparation of the manuscript. The focus on upregulation of genes stems from the specific hypotheses that were evaluated in the context of ORG treatment over CONV.

**Comment #6**: Conclusion section is also long, this must not be more than 150-200 words.
**Response**: As per the instructions to authors, there is no word limit for the conclusion section. The only requirement is that the language should be clear and concise. In the absence of any specific suggestions, the authors feel that the conclusion as written appropriately sums up all the aspects of the study clearly and concisely.

**Comment #7**: Probably the raw data of transcriptome has not been uploaded to any publicly available platform such as NCBI or any other, please upload raw data and mention accession number in the manuscript.
**Response**: In the material and methods section, under the subsection Illumina Sequencing the raw data has been described as being uploaded to the NCBI SRA and the accession numbers were included.

**Comment #8**: This article may be cited in the discussion as this is closely related with the current study "19. Muhammad Azher Nawaz, Chen Chen, Fareeha Shireen, Zuhua Zheng, Hamza Sohail, Muhammad Afzal, Muhammad Amjad Ali, Bie Zhilong, Yuan Huang. 2018. Genome-wide expression profiling of leaves and root of watermelon in response to low nitrogen. BMC Genomics. 19: 456 " <https://bmcgenomics.biomedcentral.com/track/pdf/10.1186/s12864-018-4856-x>

**Response**: Thank you for the suggestion. The published article is pertinent to this work and has been cited in the discussion.
