# Supplementary material for: Concomitant phytonutrient and transcriptome analysis of mature fruit and leaf tissues of tomato (Solanum lycopersicum L. cv. Oregon Spring) grown using organic and conventional fertilizer
Source: PLoS One. 2020 Jan 13;15(1):e0227429. doi: 10.1371/journal.pone.0227429 (PMC6957345; doi:10.1371/journal.pone.0227429)
Supplement: Attachment — Submitted filename: Response to Reviewer_Sharpe et al 12052019.docx [file pone.0227429.s022.docx]

December 5, 2019

Dear Editor,

The authors would like to thank the reviewer for their critical, and very helpful comments. We have incorporated major revisions to reduce the size of the manuscript and have also incorporated the log2fold-change data for gene expression. All the edits are visible in the manuscript with track changes. It is our hope that the manuscript will be acceptable for publication in its revised form. As advised, we have provided a point-by-point response to the reviewers’ comments. The author’s responses to reviewers’ comments are as follows.

**Reviewer Comment #1**: You have not followed the suggestions, and merely changed a few words in the revised version. We understand there is no page limit for PLOSONE articles, but it dose not means authors should write an article of 50 or 70 or 80 pages. Please reduce the length of the document, as for as conclusion section is concerned that should look like a "Conclusion". Reduce it to nearly 150-200 words written in a single paragraph. Please see some previously published articles to understand what does "conclusion" means?

**Author’s Response**: The manuscript has been substantially shortened in length. The conclusion section is now 195 words long, and presented as a single paragraph.

**Reviewer Comment** **#2**: Represent genes expression data in Log2fold change form.

**Author’s Response**: The gene expression data has now been presented in Log2Fold-change form both in the text as well as in the tables accompanying the revised figures.
